# Interactions of Mucomimetic Polymers and Meibomian Surface Films upon Exposure to Environmental Stressors

**DOI:** 10.3390/biom16081094

**Published:** 2026-07-27

**Authors:** Georgi As. Georgiev, Norihiko Yokoi, Florence Kim, Mihaela Bacheva, Miho Nishiyama, Toshiyuki Hotta

**Affiliations:** 1 Faculty of Physics, Sofia University “St. Kliment Ohridski”, 1164 Sofia, Bulgaria; mdbacheva@uni-sofia.bg; 2 Center of Competence “Clean Technologies for Sustainable Environment—Waters, Wastes, Energy for Circular Economy”, Sofia University “St. Kliment Ohridski”, 1164 Sofia, Bulgaria; 3 Department of Ophthalmology, Kyoto Prefectural University of Medicine, Kyoto 602-8566, Japan; nyokoi@koto.kpu-m.ac.jp; 4 Research and Development Department, Rohto Pharmaceutical Co., Ltd., Osaka 544-0012, Japan; florence@rohto.co.jp (F.K.); miho@rohto.co.jp (M.N.); hotta@rohto.co.jp (T.H.)

**Keywords:** relative humidity, PM2.5, tear film, tear-mimetic nanoemulsion, meibum surface properties, environmental stressors, Volmer equation-based version of 2D-virial equation of state, Maxwell viscoelastic and diffusion-relaxation model of stress relaxations

## Abstract

Environmental stressors like low temperature, low relative humidity (RH), and particulate matter (PM2.5), promote tear film instability and dry eye disease. This study investigates how these conditions alter the interfacial behavior of meibomian gland secretion (MGS) films in vitro and evaluates the capacity of mucomimetic polymers (0.5% hyaluronic acid [HA], polyvinylpyrrolidone [PVP], and chondroitin sulfate [CHS]) to suppress these impacts. MGS films over polymer-containing aqueous subphases were analyzed using a Langmuir trough and Brewster angle microscopy under adverse conditions (20 °C subphase, 20% RH, PM2.5 exposure). A sophisticated analytical framework was developed to evaluate MGS duplex multilayers: (i) a Volmer equation-based 2D-VES model to probe interfacial molecular properties (limiting area, compressibility, cohesion pressure) and (ii) a combined Maxwell viscoelastic and diffusion-relaxation model to quantify the dilatational relaxation modulus. Results indicate that despite their distinct nature, environmental stressors similarly disrupt the multilayer structure, reorganization, and rheological properties of MGS layers during blink-like deformations. Polymer supplementation moderated these adverse effects, yielding partial recovery of film structure and isothermal reversibility. Distinct mechanisms of action for HA, PVP, and CHS at the film/aqueous interface are elucidated.

## 1. Introduction

The boundary between the atmosphere and the aqueous phase of the tear film is stabilized by the tear film lipid layer (TFLL), a viscoelastic duplex architecture with a thickness of approximately 100 nm [1,2]. Structurally, it comprises a basal monolayer of amphiphilic polar lipids (PL) in direct contact with the aqueous tear (AT), overlaid by a thicker, exterior bulk phase-like stratum of nonpolar lipids (NPL) [3,4]. This protective barrier is primarily composed of meibum (MGS)—the secretory output of the meibomian glands. Chemically, this secretion is dominated by NPL (exceeding 90%), including various triglycerides, wax esters, and cholesteryl esters, whereas the PL fraction (under 10%) is characterized largely by (O-acyl)-ω-hydroxy fatty acids [5,6].

The integrity of the tear film is frequently undermined by adverse external conditions, such as depressed ambient temperatures, low relative humidity (RH), and the presence of fine particulate matter (PM2.5). These stressors are recognized drivers in the pathogenesis of dry eye disease (DED) [7,8,9,10]. As the most superficial component of the ocular surface, the tear film lipid layer (TFLL) serves as the primary shield against such environmental challenges. As we recently showed, despite their distinct nature, these adverse ambient conditions appear to influence MGS films in a similar fashion, by disturbing the uniform spreading, the morphological integrity, and the viscoelastic properties of meibum [11]. Indeed, decreased RH is expected to facilitate the evaporative cooling of TFLL (due to the AT exsiccation to the air), thus sharing a common physical mechanism with the impact of low environmental temperature.

Polyanionic mucomimetic polymers, such as hyaluronic acid (HA), polyvinylpyrrolidone (PVP), and chondroitin sulfate (CHS), play a sophisticated role in stabilizing the tear film lipid layer (TFLL) by enhancing its structural integrity and spreading dynamics. Rather than merely increasing bulk viscosity, these polymers—particularly the high-molecular-weight variants of HA—interact with the polar lipid subphase (e.g., phospholipids) to reinforce the interfacial “scaffold” upon which the non-polar lipids reside [12,13,14].

Research indicates that these polymers can integrate into the lipid-aqueous interface, reducing surface tension and promoting the formation of a more cohesive and continuous lipid film during the blink cycle in vitro and in vivo [12,13,14,15,16]. This interaction mitigates the formation of “lipid-free” gaps, thereby effectively reducing the rate of aqueous evaporation and increasing tear film break-up time (TFBUT). Furthermore, the mucomimetic properties of CHS and HA allow for superior retention on the ocular surface through non-covalent bonding with membrane-associated mucins, providing a synergistic lubricating effect that minimizes friction-induced damage to the corneal epithelium [12,13,14].

The primary goal of this investigation is to decipher the synergistic relationship between mucomimetic agents—specifically hyaluronic acid (HA), polyvinylpyrrolidone (PVP), and chondroitin sulfate (CHS)—and the meibomian gland secretion (MGS). We seek to determine whether these polymers stabilize the tear film’s lipid component when subjected to environmental stressors, such as particulate matter (PM2.5) contamination, reduced thermal states (20 °C), and arid conditions (20% relative humidity). Gaining insight into the interplay among these molecules at the air–liquid interface is essential for identifying the biophysical pathways through which the polymers can shield the ocular surface from instability. To probe these complex interactions, we utilized a Langmuir trough to generate artificial tear film models [12,17,18,19,20]. The properties of these interfacial layers were analyzed both in isolation and when formed over the polymer-enriched subphase via analysis of their surface pressure area isocycles, dilatational rheology (probed with stress relaxation technique) and film morphology visualized with Brewster Angle Microscopy (BAM).

## 2. Materials and Methods

### 2.1. Experimental Methods

Surface pressure-area (π-A) isotherms were recorded using a µ Trough XS Langmuir surface balance (Kibron, Helsinki, Finland) equipped with a Wilhelmy wire probe (sensitivity: 0.01 mN/m). The trough featured a total surface area of 135 cm^2^ and a 100 mL subphase volume. All measurements were conducted at a pH of 7.4 using phosphate-buffered saline (PBS) as the subphase. To maintain environmental integrity and minimize evaporation of the phosphate-buffered saline (PBS) subphase, the experimental setup was enclosed within an acrylic housing.

The films are formed by spreading with a Hamilton microsyringe of 20.3 micrograms MGS (collected from healthy human volunteers as previously described [11,12]) from the chloroform solution over PBS subphase, pure and at 0.5% concentration of polymers (mean molecular weight of the polymers is 1000 kDa; all provided by Rhoto Pharmaceutticals, Osaka, Japan) dissolved into it after the MGS film was deposited at the air/water surface. For the experiments with PM2.5 the airborne particulate matter was premixed with the MGS solution in CHCl_3_ to a 30 wt% content. A 15 min equilibration period was observed to allow for complete solvent (chloroform) evaporation. Subsequently, the interfacial film was subjected to dynamic compression–expansion cycles via bilateral barrier movement at a constant velocity of 70 mm/min, ensuring film containment throughout the process. Data acquisition focused on the “stationary” π(A) isotherms obtained after the third cycle to ensure thermodynamic stability. Experimental reproducibility was confirmed through triplicate measurements, demonstrating a deviation of less than 2% between isotherms. Interfacial morphology and film structure were characterized in situ using Brewster angle microscopy (MC-BAM, Imperx, Boca Raton, FL, USA).

Under standard experimental conditions, the aqueous subphase was maintained at 35 °C, simulating the surface temperature of a healthy cornea in a moderate climate, with a relative humidity (RH) of 80%. While the RH of 40–60% is commonly reported in office conditions, these values had no impact on the surface properties of the meibomian samples compared to 80% RH. Thus, as explained elsewhere [11,14,17,18,19,20] 80% RH was preferred for these experiments as it allowed for better suppression of aqueous subphase evaporation when extended transients of surface pressure were measured.

To evaluate the impact of environmental stressors [8,9,10,21], three specific conditions were tested:

(1) *Cold Stress*: The temperature was lowered to 20 °C (reflecting the cooling of tears in winter conditions) at 80% RH [22,23,24].
(2) *Evaporative Stress*: The RH in a room with controlled humidity was reduced to 20% at 35 °C. For these trials, the sample was equilibrated at low humidity for 30 min before beginning the compression/expansion isocycles. During the experiments, an infusion pump was utilized to supplement the aqueous subphase at a rate of 10 µL/min. This constant replenishment, delivered behind the barriers of the Langmuir trough, compensated for evaporative loss and ensured a stable subphase volume throughout the measurements.
(3) *Particulate Stress*: Atmospheric pollution was simulated at 35 °C and 80% RH by introducing PM2.5 material (ERM^®^-CZ110; Sigma-Aldrich, St. Louis, Missoury, USA) at a concentration of 30 wt% relative to the Meibomian Gland Secretion (MGS).

To estimate the dilatational viscoelastic properties of the films, a minor compression step of ∆A/Ao = 5% ± 1% (where Ao—initial film area, ∆A—applied change) was applied. This step change was executed once the film had equilibrated at an initial surface pressure of π_0_ = 10 mN/m [25,26]. The resulting surface pressure relaxation transients ∆π(t) relaxation transients were then recorded and analyzed.

### 2.2. Theoretical Apparatus: Analysis of Surface Pressure-Area Isotherms

**Figure 1:**
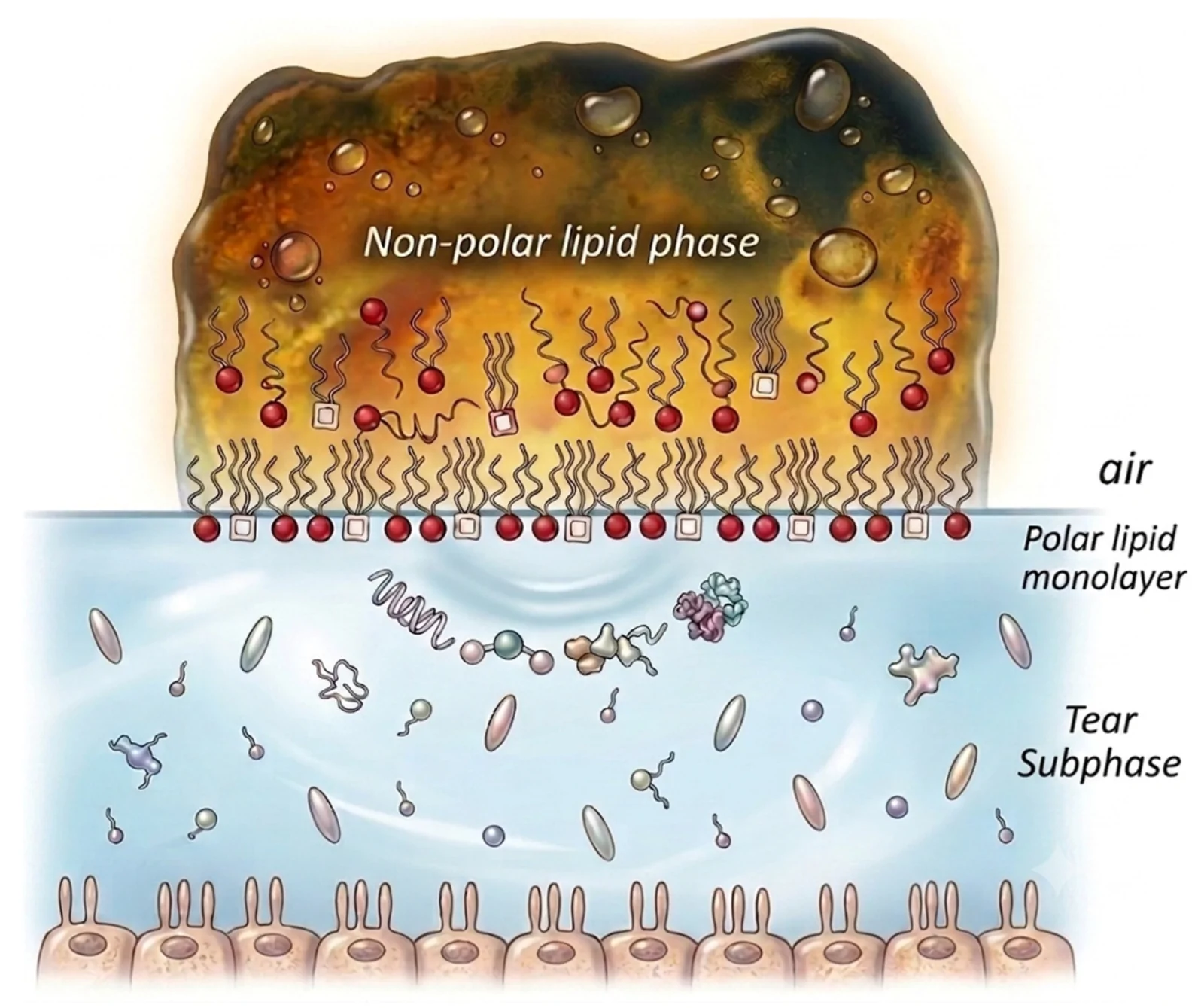
Simplified illustrative scheme of MGS duplex films spread over subphase of aqueous tear (a composite saline solution of secretory mucin, proteins, and some polar lipids) and underlying corneal epithelium cells (adapted from [1,12] where further explanations regarding the sketch can be found).

Due to their extreme hydrophobicity, instead of spreading as monolayers meibomian samples spontaneously form multilayers (Figure 1) at the air/water interface even at zero surface pressure:

Due to their duplex structure the highly valuable information regarding what type of molecules are present at the meibomian (MGS) film interface (in health and diseases, in presence of polymers, at various ambient conditions, etc.) with aqueous subphase is impossible to be directly calculated. Although the amount of meibum deposited on the aqueous surface of the Langmuir trough is known, it does not allow answering the question in mind, as due to its extreme hydrophobicity MGS is inherently multilayer forming. Thus, in our and other studies it turns out that, upon maximum compression, the area per molecule [if using the classic simple relation where *area per molecule equals the ratio of (area of the trough/number of molecules)*] gives something like 4 Å^2^ while the own intrinsic area per lipid molecule is 20–40 Å^2^. Thus, obviously direct calculation is irrelevant and produces physically absurd estimates. On top of this, to estimate the number of molecules, one has to inevitably attribute some mean molecular weight to MGS, which is another obvious limitation, considering the complex multicomponent nature of meibum. While in the context of MGS the issue has not been addressed until now, it is a problem that has been encountered in other aspects of surface chemistry research. The problem first appeared in Langmuir surface balance studies of sea surface microlayers (a good review is provided in reference [27]).

In these studies, the surface pressure area compression isotherm is described via a version of the two-dimensional virial equation of state (2D-VES):

(1) $$\pi A=C_{0}+C_{1}\pi +C_{2}\pi^{2}$$

where *C*_0_, *C*_1_, *C*_2_ are the virial coefficients, and *A* is the *total film area* (in cm^2^). *C*_1_ can be interpreted as the limiting specific area occupied by the molecules in the film, and *C*_0_ can be assumed equal to:

(2) $$C_{0}=XnKT$$

where *n* is the number of molecules in the unknown film, *K* is the Boltzmann constant, *T* is temperature and *X* is the molecular aggregation factor. If *X* = 1, we then have an ideal case where there is no attraction or repulsion between molecules. This ideal case, however, does not exist in the real world. Thus, to use Equation (2) for calculation of the number of molecules in the film, it is thought to be necessary to assume a value for the interaction parameter *X*. In the sea surface microlayers research, the value proposed by Barger and Means [28] *X* = 0.5 (±0.75) is used. Although this approach was adopted in a number of studies, there is the obvious problem that (i) the value of *X* comes with huge uncertainty and that (ii) while *X* = 0.5 might be acceptable for surface layers from water-soluble polymers, it is highly inaccurate when lipid films are considered where X ≤ 0.05 are frequently observed [29]. Thus, although Equations (1) and (2) have been commonly implemented, a more precise and assumption-free approach is called for.

In the 1970s, Taylor and Kaizer [30] developed an alternative formulation of 2D-VES for “liquid extended” films with surface pressure π ≤ 10 mN/m

(3) $$\pi A=nRT+\pi A_{00}-\pi^{2}kA_{00}$$

Here *A*_00_ = *nA_limiting_* is the measure for the intrinsic molecular area of the molecules in the surface film and *k* is the molecular compressibility constant.

Looking at Equation (3), one can see that it is a form of Equation (1) where in-depth physical meaning is ascribed to the various coefficients of 2D-VES. Looking at the first term of the right-hand side of equality, it can be seen that in Equation (3) an assumption is made that *X* = 1. This in turn posed an important challenge for the application of Equation (3) as it was necessary to find a surface pressure range where this condition is reasonably obeyed. A detailed procedure was developed: a plot is made of (*π*_2_*A*_2_ − *π*_1_*A*_1_) vs. *π*_1_, where, typically, (π_2_ − π_1_) = 1 mN/m and, whenever this plot is linear, Equation (1) is applicable. Then, the parameters *n*, *k*, and *A*_00_ can be determined by fitting of the surface pressure area isotherms with Equation (3). This procedure made the application of Equation (3) cumbersome and although it found its way into a number of high-quality studies for determination of the apparent number of molecules (and related properties) in unknown undefined surface films [31] the adoption of Equation (3) remained relatively limited.

An important and overlooked opportunity to improve upon the limitations of the equations considered up to now is offered by the Volmer equation of state for “liquid expanded” (i.e., π = 0–10 mN/m) surface film developed by V. B. Fainerman (see ref. [32] for review):

(4) $$\pi =\frac{KT}{A_{m}-A_{lim}\left(1-k\pi \right)}-\pi_{coh}$$

Here, *A_m_* is the area per molecule and it can be related with *n* (the apparent number of molecules at the aqueous interface) as follows

*A* = *nA_m_* and *A*_00_ = *nA_lim_* (4a)

where *A* is the total film area in cm^2^ (i.e, the area of the trough). $\pi_{coh}$ is the cohesive surface pressure characteristic for the cohesion between the molecules and thus for their resistance to spread. Using Equations (4) and (4a) it turned out to be possible to fit experimental surface pressure area isotherms and to obtain reasonable estimates for the apparent number of molecules, mean molecular area and molecular compressibility of lipid-containing films with undefined composition [33,34].

What was overlooked in these studies is that, using the relationships explained up to now, it is possible to rearrange Equations (4) and (4a) into the form of 2D-VES

(5) $$\pi A=nKT\frac{\pi }{\left(\pi +\pi_{coh}\right)}+\pi A_{00}-\pi^{2}kA_{00}$$

This is 2D-VES from Equation (1) rewritten in such a manner that there is concrete physical meaning to each of the coefficients of the equation and where the aggregation factor *X* no longer needs to be assumed, as the equation shows a physically meaningful way to represent it:

(5a) $$X=\frac{\pi }{\left(\pi +\pi_{coh}\right)}$$

As the monolayer reciprocal compressibility modulus is

*C_s_*^−1^ = *A_π_* (−*dπ*/*dA*) (6)

by taking the first derivative *dπ*/*dA* from Equation (4), one obtains:

(7) $$C_{s}^{-1}=\frac{\left(\pi +\pi_{coh}\right)\left(1+\left(\frac{A_{lim}}{KT}\right)\left(1-k\pi \right)\left(\pi +\pi_{coh}\right)\right)}{1+k\left(A_{lim}/KT\right){\left(\pi +\pi_{coh}\right)}^{2}}$$

Using Equation (7) it is possible to obtain unique values for the molecular compressibility *k*, for *A_lim_* (where the relation *A*_00_ *= nA_lim_* is valid) and for $\pi_{coh}$ which allows these estimates to be used in Equation (5) (or to use them in a constrained optimization fit) and to obtain a unique solution for the apparent number of molecules *n* at the aqueous surface. Thus, we reach an assumption-free solution of the question set in the beginning of this subsection, based entirely on surface chemistry data and free of any speculative simplifying assumptions.

In addition to the calculations explained above, for surface pressure/area compression/expansion isocycles the isothermal reversibility is calculated using Equation (8) [35]:

(8) $$Rv=100\frac{{\left(\int_{A_{i}}^{A_{f}}\pi dA\right)}_{expansion}}{{\left(\int_{A_{i}}^{A_{f}}\pi dA\right)}_{compression}}\%$$

### 2.3. Theoretical Apparatus: Analysis of Stress Relaxation Transients

For multilayer films stress relaxations can be described with combined Maxwell viscoelastic and diffusion-relaxation model [36]:

(9) $$\Delta \pi \left(t\right)=\Delta \pi_{inf}+A_{M}\exp\left(\frac{-t}{\tau_{M}}\right)+A_{D}\exp\left(\frac{2t}{\tau_{D}}\right)erfc\sqrt{\left(\frac{2t}{\tau_{D}}\right)}$$

where the first two terms on the right account for viscoelastic contribution in the relaxation of an interfacial layer and the last term reflects diffusion of active species from a polar lipid surface interface into the bulk layer during compression and diffusion in the other direction when the film is expanded. *τ_M_* and *τ_D_* represent characteristic times for viscoelastic relaxation and diffusive exchange, respectively. A_M_ and A_D_ are constants characterizing the relative contributions of viscoelastic and diffusion mechanisms into the stress relaxation transient, respectively.

The problem with this equation in its original form is that due to the presence of five fitting parameters it hardly converges to a unique solution which limited its broader adoption in research practice. However, using the mathematics of exponential decay kinetics [37,38] Equation (9) can be rearranged as follows:

(10) $$\Delta \pi \left(t\right)=\Delta \pi_{inf}+[\Delta \pi_{max}-\Delta \pi_{inf}]\;[A_{M}\exp\left(\frac{-t}{\tau_{M}}\right)+\left(1-A_{M}\right)\exp\left(\frac{2t}{\tau_{D}}\right)erfc\sqrt{\left(\frac{2t}{\tau_{D}}\right)}]$$

where is $\Delta \pi_{max}$ the maximum surface pressure increment from which the relaxation starts (it is taken directly from the experimental data); $A_{D}=$ $\left(1-A_{M}\right)$; and $A_{D}+A_{M}=1$. In Equation (10), there are only four fitting parameters subject to the constraints defined above which allow for Equation (10) to converge to a unique solution.

## 3. Results

### 3.1. Surface Properties of Meibum Polymer Films at Normal Ambient Conditions: 35 °C, 80% RH

**Figure 2:**
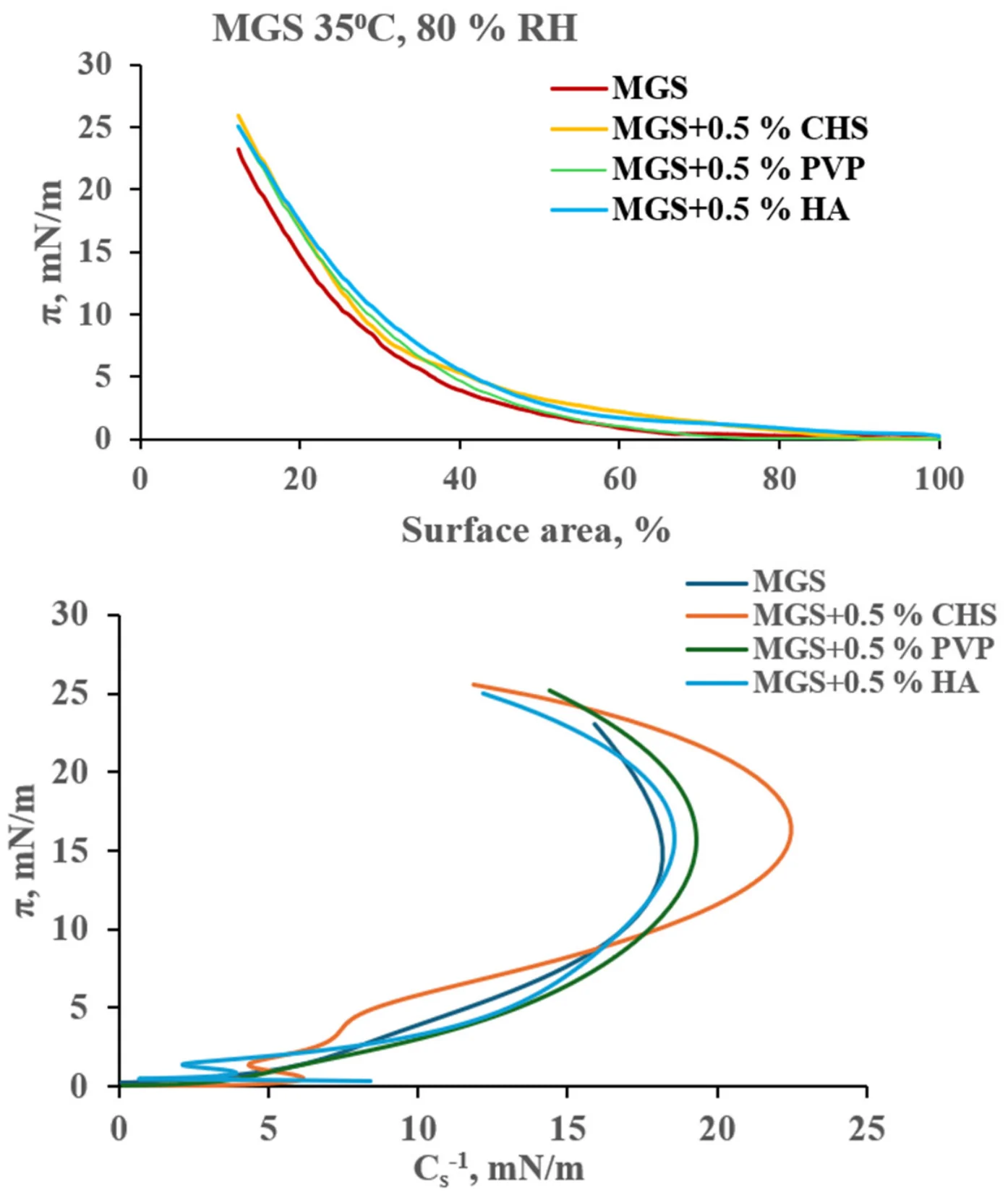
Surface pressure/area isotherms of MGS, pure and upon inclusion of 0.5% mucomimetic polymers in the aqueous subphase (**upper panel**) and of the isothermal reciprocal compressibility modulus/surface pressure dependencies (**lower panel**).

**Figure 3:**
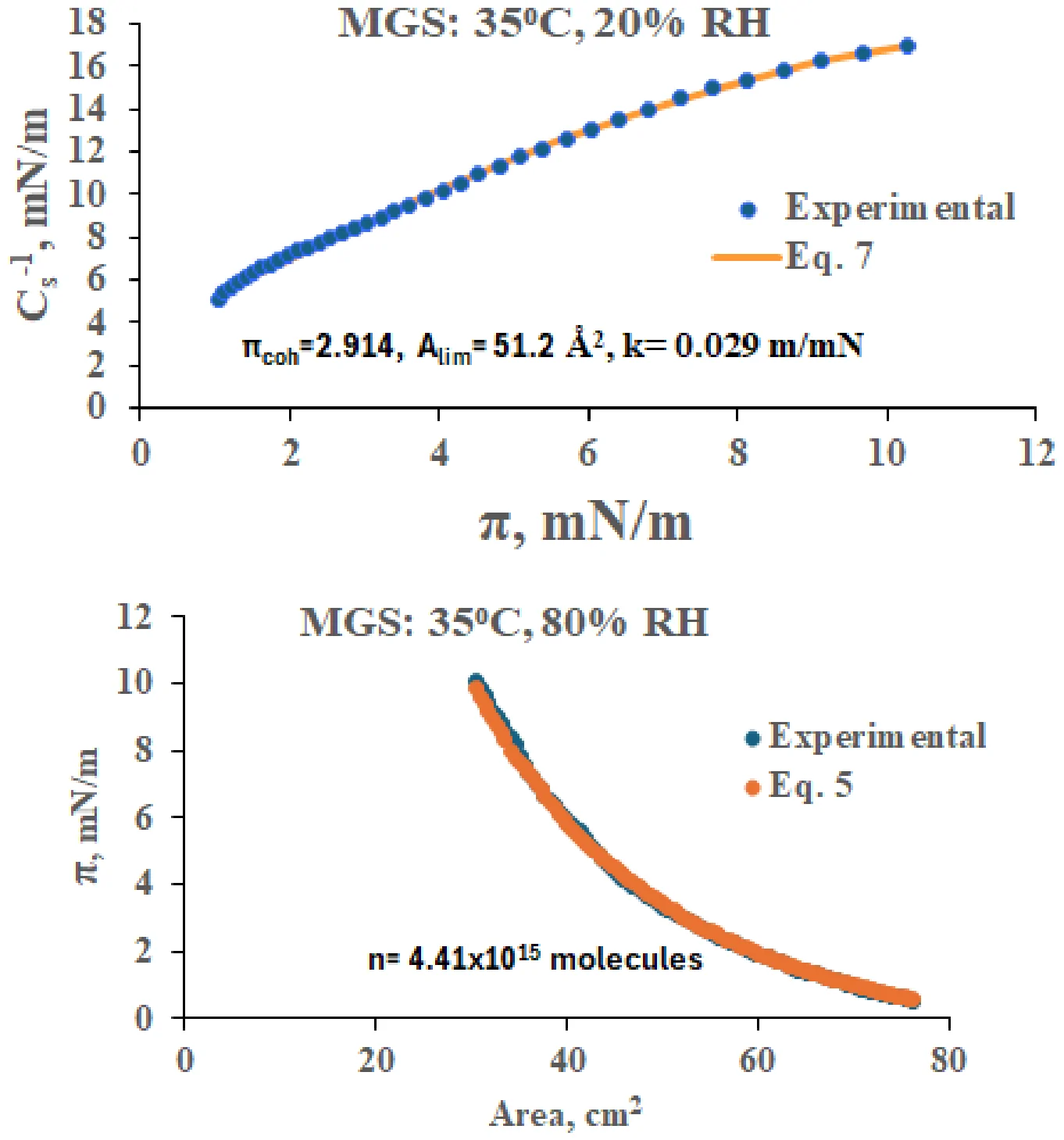
Fitting (R^2^ = 0.99) of *C_s_^−^*^1^ (π) (**upper panel**) and π(A) (**lower panel**) data with Equations (5) and (7) makes it possible to obtain data for the apparent number of lipid molecules, molecular compressibility, limiting molecular area, and cohesive surface pressure of MGS films. For all experimental data in the study, the quality of the fit with Equations (5) and (7) is always R^2^ ≥ 0.98.

Surface pressure/area data are summarized at Figure 2. As reported elsewhere [20] at normal ambient conditions MGS shows high isothermal reversibility (Rv = 78.34%) alone and in the presence of mucomimetic polymers. The inclusion of the polymers resulted in a slight (2–3 mN/m) shift of the compression isotherms toward higher surface pressure values. As revealed by the analysis of the isothermal reciprocal compressibility modulus (Equation (6); Figure 2 (lower panel)) *C_s_^−^*^1^ remained < 50 mN/m over the entire π range, suggesting liquid-extended-like molecular packing of the MGS layers at the aqueous interface. The data at Figure 2 were successfully analyzed with Equations (5) and (7), through which excellent quality of fit (R^2^ ≥ 0.98 for all the experimental data in the study) was obtained (Figure 3). The values obtained for apparent number of lipid molecules, molecular compressibility, limiting molecular area, and cohesive surface pressure of MGS films, alone or in the presence of 0.5% mucomimetic polymers are summarized in Table 1. It can be seen (Table 1) that the inclusion of polymers resulted in more compressible (higher value of *k*) films and in a drop of *π_coh_* (i.e., improved spreading) which is typical for polymer-containing films. In line with this (with the exception of CHS), the presence of PVP resulted in an increase of the apparent values of *A_lim_* and decrease of the apparent number of molecules at the film/aqueous interface, which is also indicative for the inclusion of polymers in the molecular packing. As per MGS and MGS + 0.5% CHS layers, the *A_lim_* are typical for different degrees of compression of OAHFA, the intrinsic amphiphilic species of MGS.

**Figure 4:**
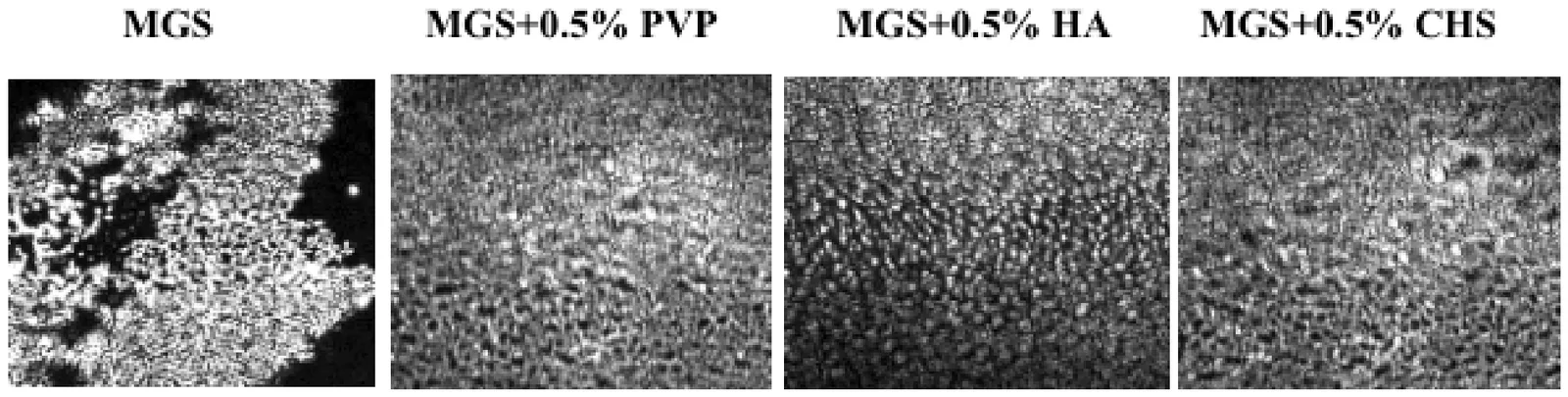
BAM micrographs (500 µm × 300 µm) of MGS layers, alone and upon inclusion of 0.5% mucomimetic polymers in the aqueous subphase, at π = 10–15 mN/m at 35 °C, 80% RH.

BAM micrographs (Figure 4) reveal the enhancement of the continuous multilayer structure of meibomian films upon supplementation of mucomimetic polymers in the films aqueous subphase.

**Figure 5:**
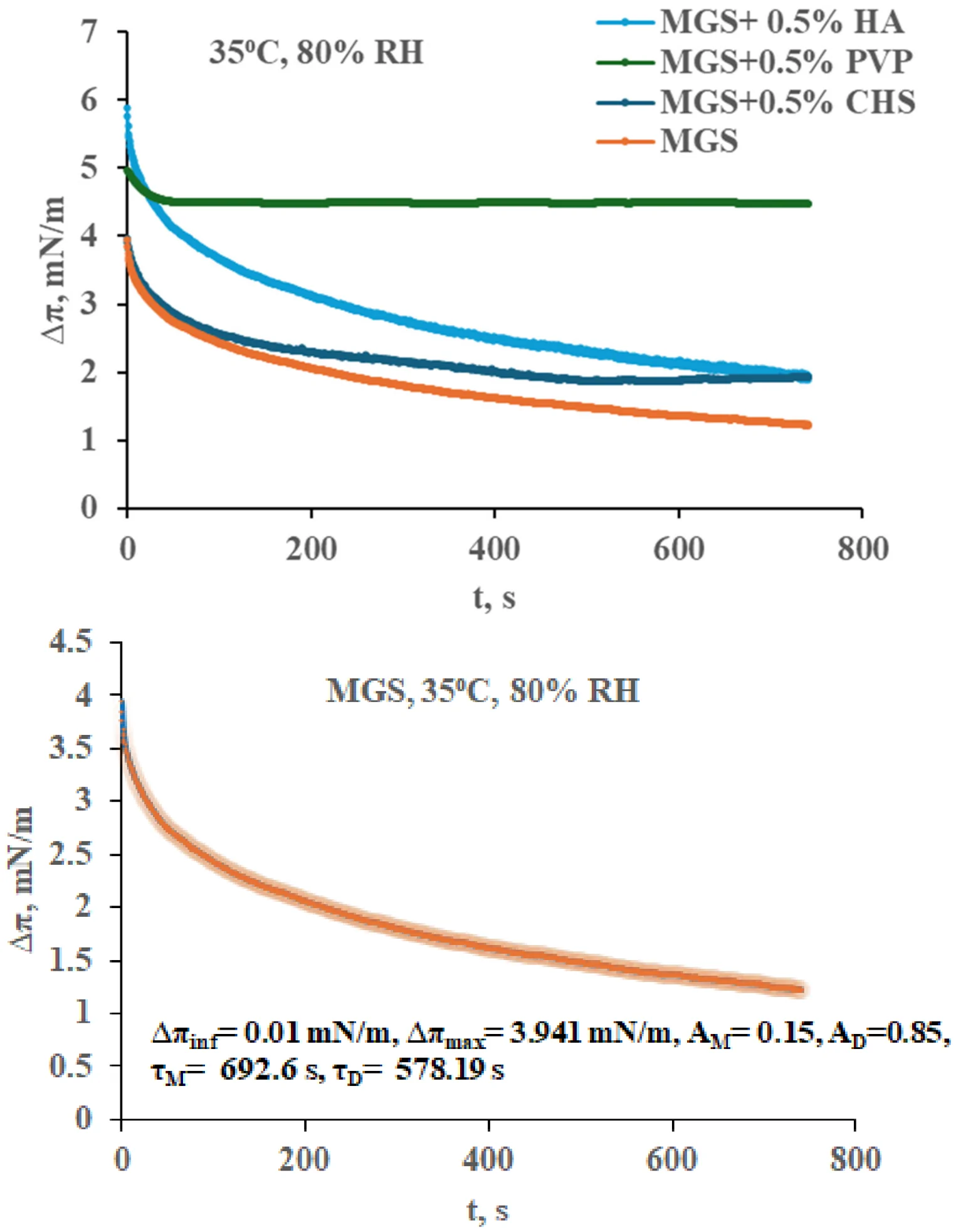
Surface pressure relaxation transients of meibomian films, pure and in the presence of 0.5% mucomimetic polymers, at 35 °C, 80% RH (**upper panel**). Representative fit (solid thin line, R^2^ = 0.99) of MGS film stress relaxation via Equation (10) is shown at the (**lower panel**).

The stress relaxation transients of the surface films are shown at Figure 5 (upper panel) and a representative fit (R^2^ = 0.99) is presented at Figure 5 (lower panel). The results from the analysis of the stress relaxations via Equation (10) are summarized in Table 2. It can be seen that the supplementation of polymers shifted the relaxation process towards higher ∆π_inf_ and changed the balance between the viscoelastic and diffusion processes with an overall raising of the contribution of viscoelasticity compared to the one of diffusional rearrangement of molecules across the strata of the MGS duplex film.

### 3.2. Surface Properties of Meibum Polymer Films at Low Temperature: 20 °C, 80% RH

**Figure 6:**
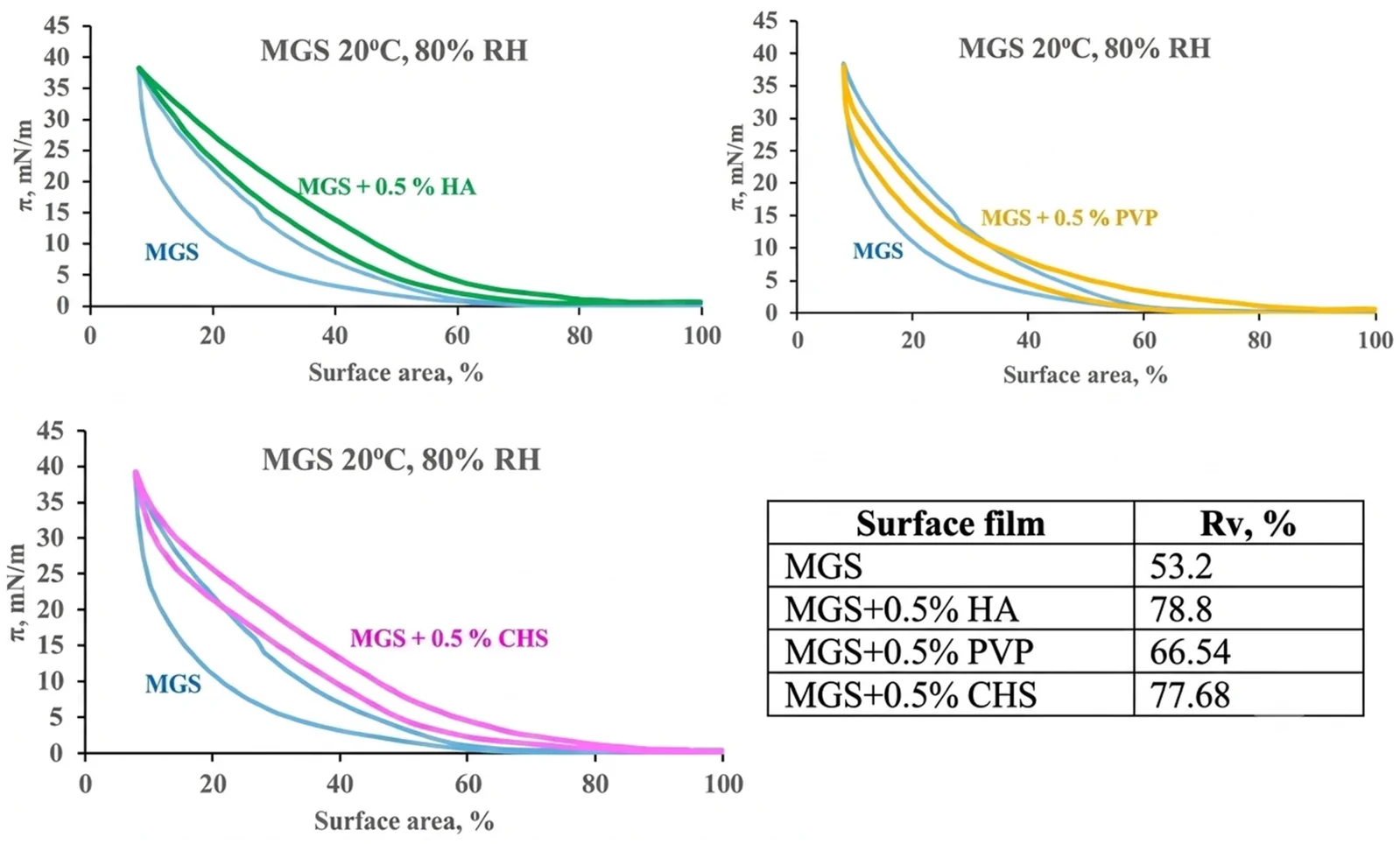
Compression–expansion π(A) isocycles of MGS films, alone and upon inclusion of 0.5% mucomimetic polymers in the aqueous subphase at 20 °C, 80% RH. The tabular insert summarizes the isothermal reversibility values as calculated with Equation (8).

Surface pressure/area isocycles are summarized at Figure 6.

**Figure 7:**
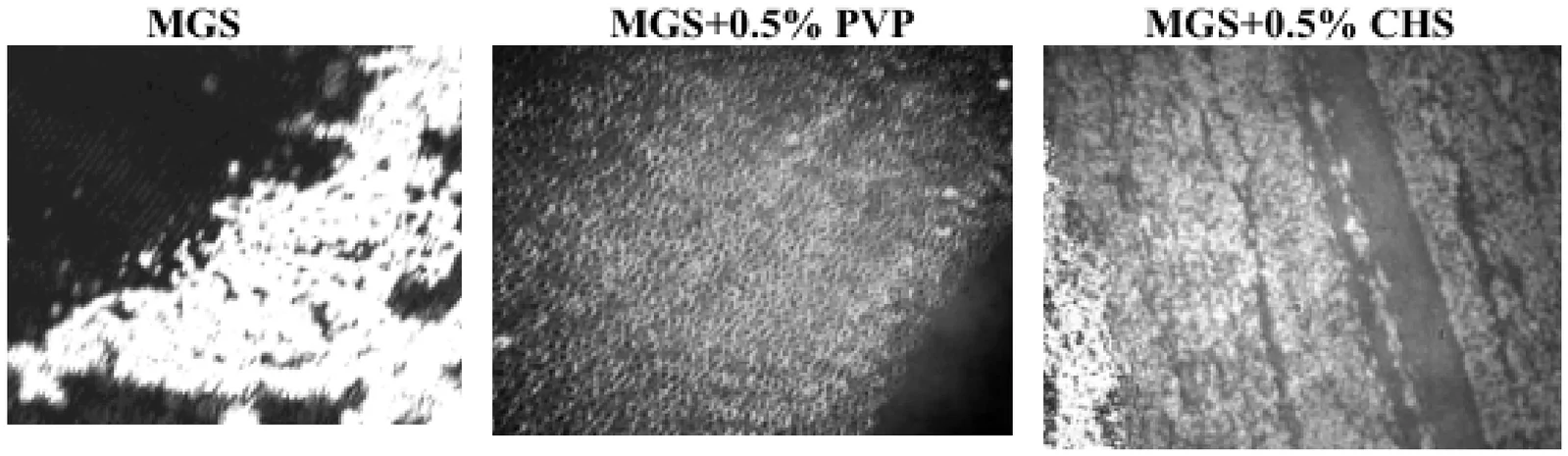
BAM micrographs (500 µm × 300 µm) of MGS layers, alone and upon inclusion of 0.5% mucomimetic polymers in the aqueous subphase, at π = 10–15 mN/m at 20 °C, 80% RH.

As previously reported, the decrease in the temperature of the aqueous subphase resulted in (i) expansion of the MGS layers (i.e., shift of the isotherms to higher surface area and higher π) due to redistribution of amphiphilic polar lipids from the non-polar stratum of the duplex film to the interface between the aqueous subphase and the MGS layer and (ii) in a drop of MGS isothermal reversibility. The latter effect is associated with worsened (re)spreading of meibomian lipids upon cyclical blink-like deformations and is known to correlate with deteriorated TFLL performance at the ocular surface in vivo. The inclusion of polymers improved the isothermal reversibility of the meibomian layers, which is also illustrated by the polymer-induced recovery of the continuous MGS layer structure as visualized by BAM (Figure 7).

**Figure 8:**
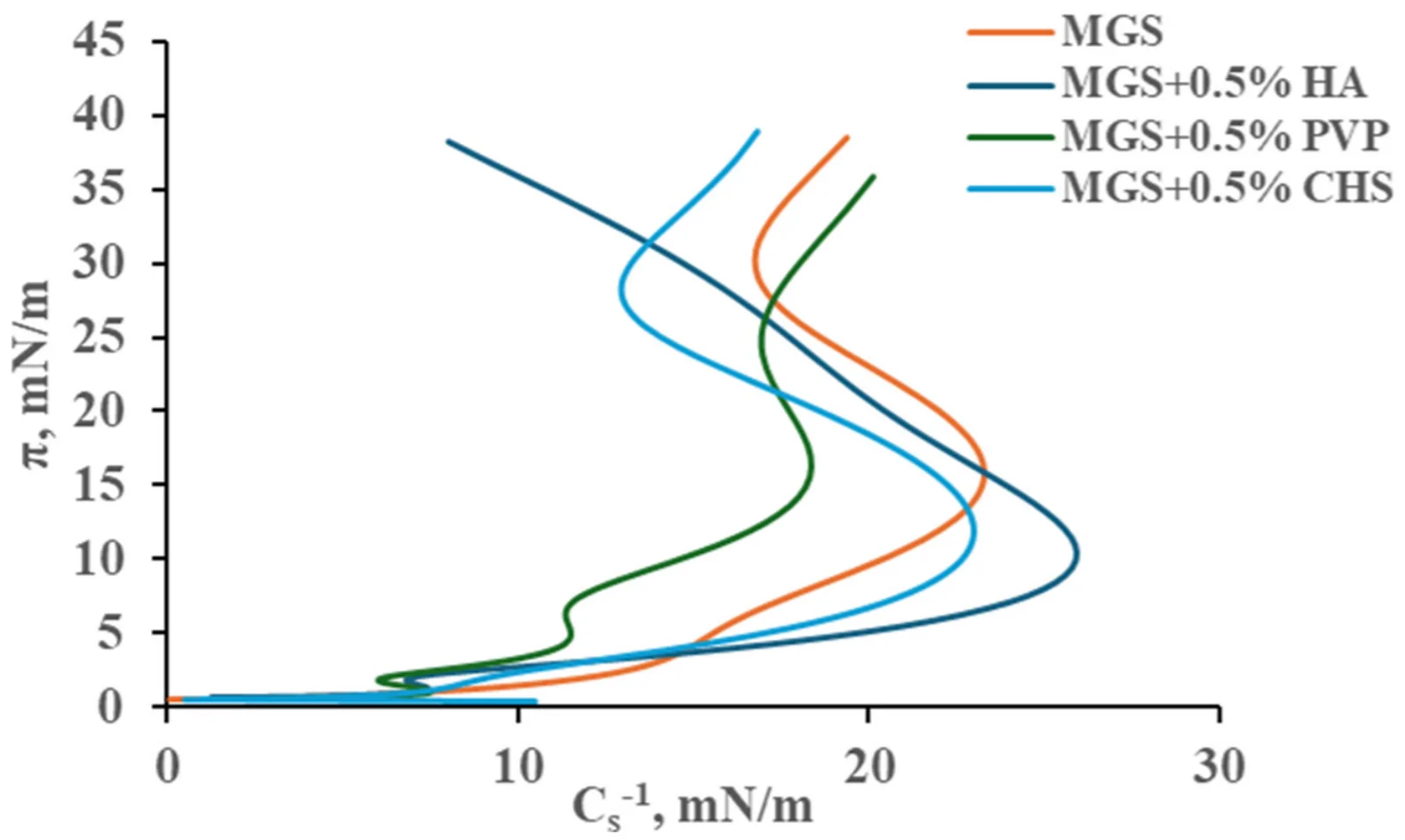
Isothermal reciprocal compressibility modulus/surface pressure dependencies of MGS layers, alone and upon inclusion of 0.5% mucomimetic polymers in the aqueous subphase, at 20 °C, 80% RH.

The *C_s_^−^*^1^ (π) curves (Figure 8) reveal that *C_s_^−^*^1^ remains lower than 50 mN/m (i.e., the films are within the liquid extended packing density) thus allowing for the application of a 2D-VES framework.

The information obtained by the analysis of the compression π(A) isotherms (Figure 6) by Equations (5) and (7) is summarized in Table 3.

Similarly to the trends at normal ambient conditions, the presence of the polymers in the subphase resulted in partial incorporation of the polymers into the surface films (reflected by the decrease in the apparent number of molecules at the film/aqueous interface and the increase in *A_lim_*) and formation of layers with enhanced compressibility and spreading (i.e., lower *π_coh_*).

**Figure 9:**
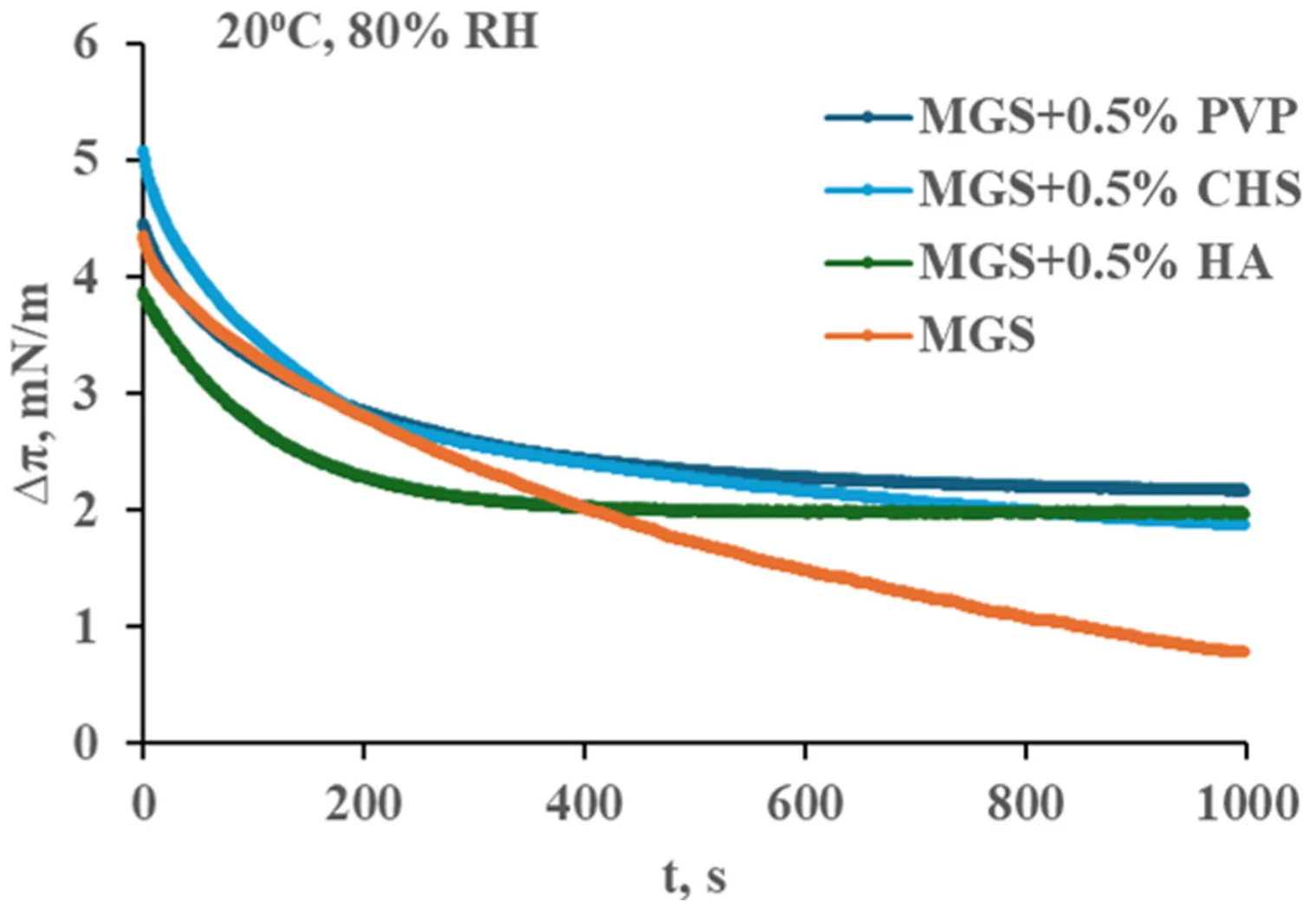
Surface pressure relaxation transients of meibomian films, pure and in the presence of 0.5% mucomimetic polymers, at 20 °C, 80% RH.

Stress relaxation transients are presented at Figure 9 and the outputs of their analysis with Equation (10) is summarized in Table 4.

The inclusion of polymers altered the relative contributions of viscoelastic and diffusion processes in the films and invariably resulted in higher ∆π_inf_, suggesting enhanced elastic response of the layers upon dilatational deformation.

### 3.3. Surface Properties of Meibum Polymer Films at Low Relative Humidity: 35 °C, 20% RH

**Figure 10:**
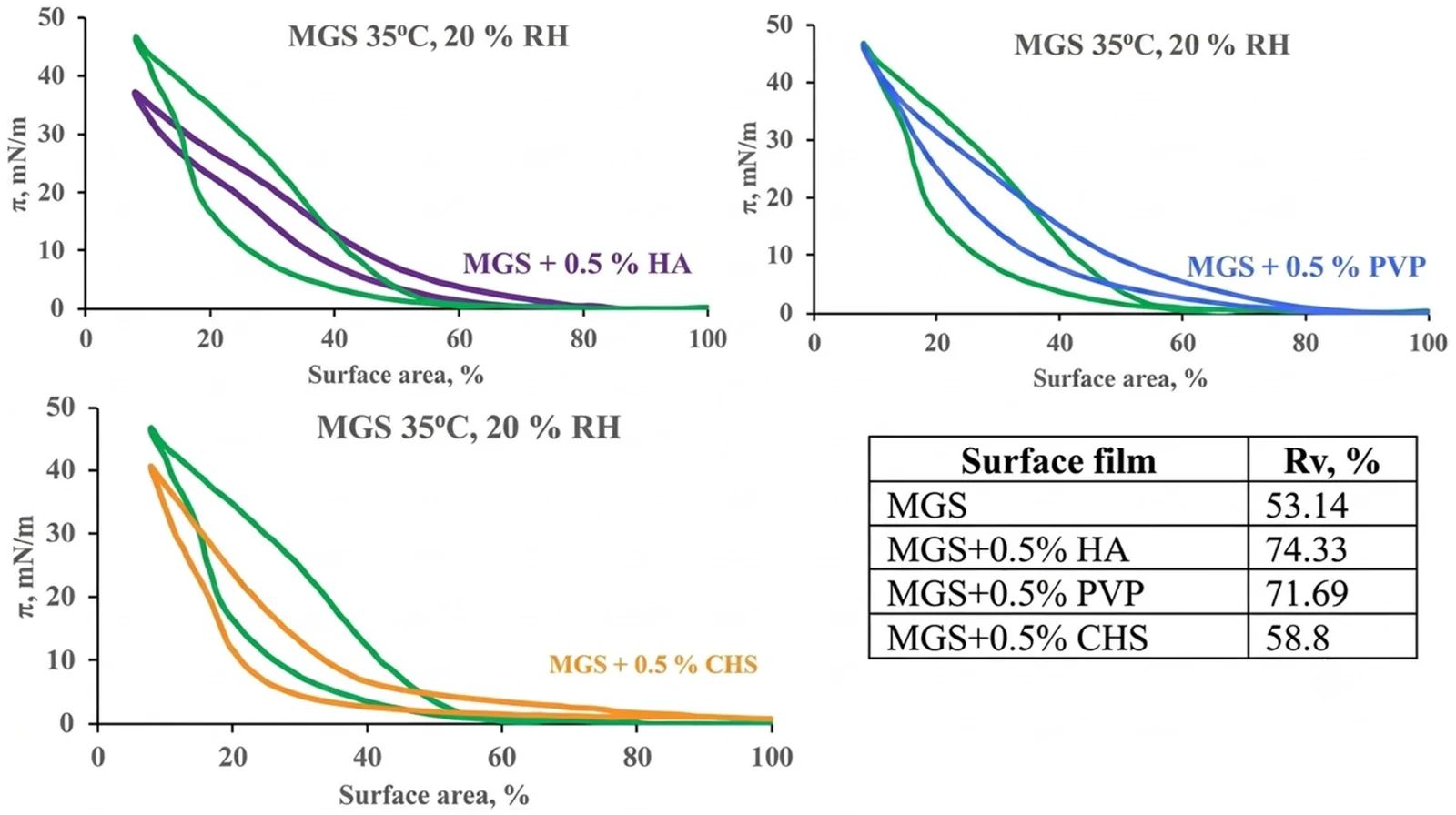
Compression–expansion π(A) isocycles of MGS films, alone (green line) and upon inclusion of 0.5% mucomimetic polymers in the aqueous subphase at 35 °C, 20% RH. The tabular insert summarizes the isothermal reversibility values as calculated with Equation (8).

**Figure 11:**
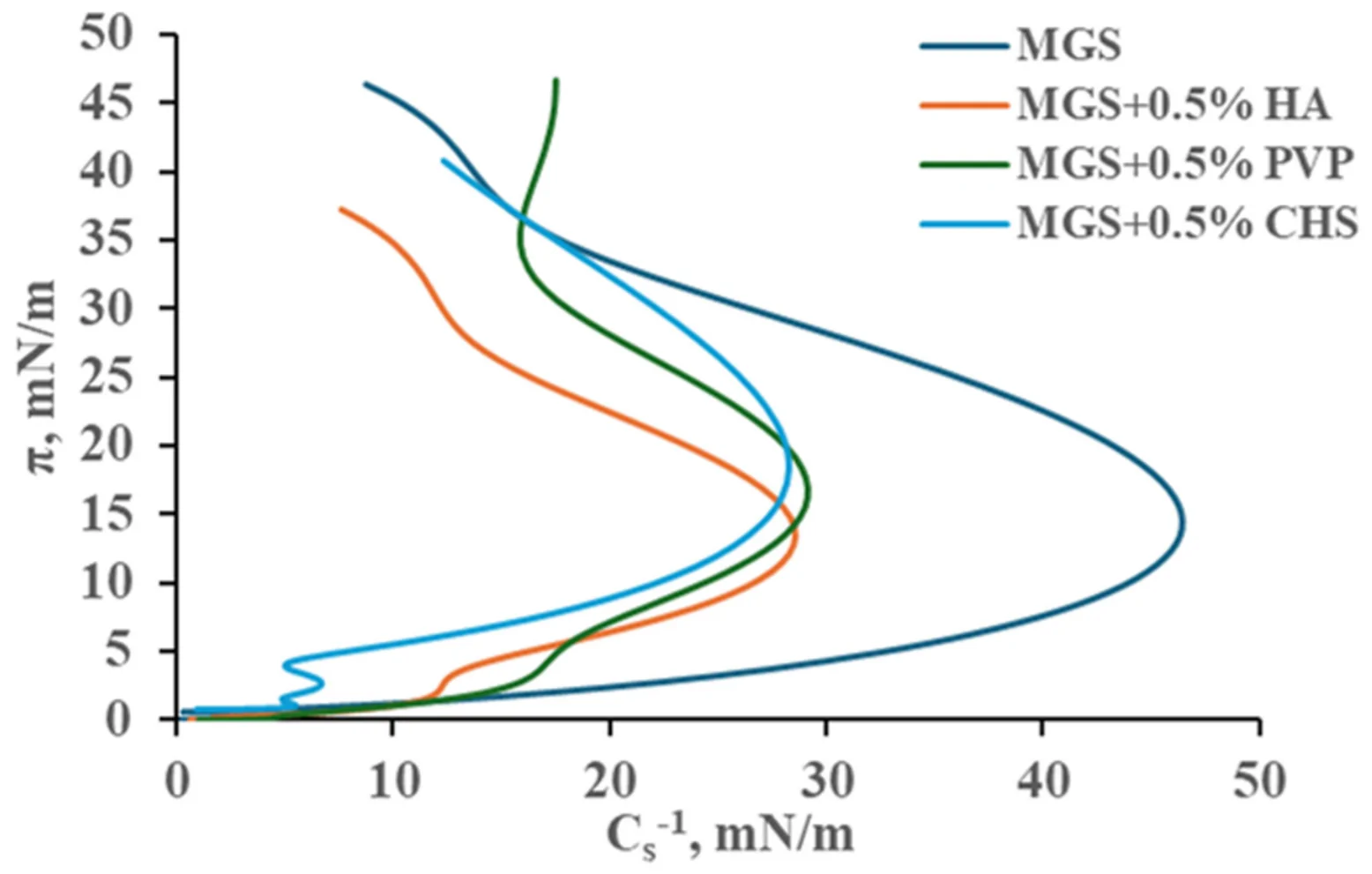
Isothermal reciprocal compressibility modulus/surface pressure dependencies of MGS layers, alone and upon inclusion of 0.5% mucomimetic polymers in the aqueous subphase, at 35 °C, 20% RH.

Similarly to the impact of low temperature, the decrease of relative humidity resulted in a drop of isothermal reversibility while shifting the π(A) isocycles towards higher surface pressure (Figure 10) compared to surface film behavior at normal ambient conditions. Here, the surface films again remain in the liquid-expanded-like state (*C_s_^−^*^1^ < 50 mN/m) where the inflexions of the *C_s_^−^*^1^ (π) curves point to complex reorganizations in the surface films at the corresponding surface pressures (Figure 11).

The analysis of the π(A) compression isotherms through Equations (5) and (7) is summarized in Table 5, showing that polymers alternate the molecular organization at the film/aqueous interface in a diverse manner.

**Figure 12:**
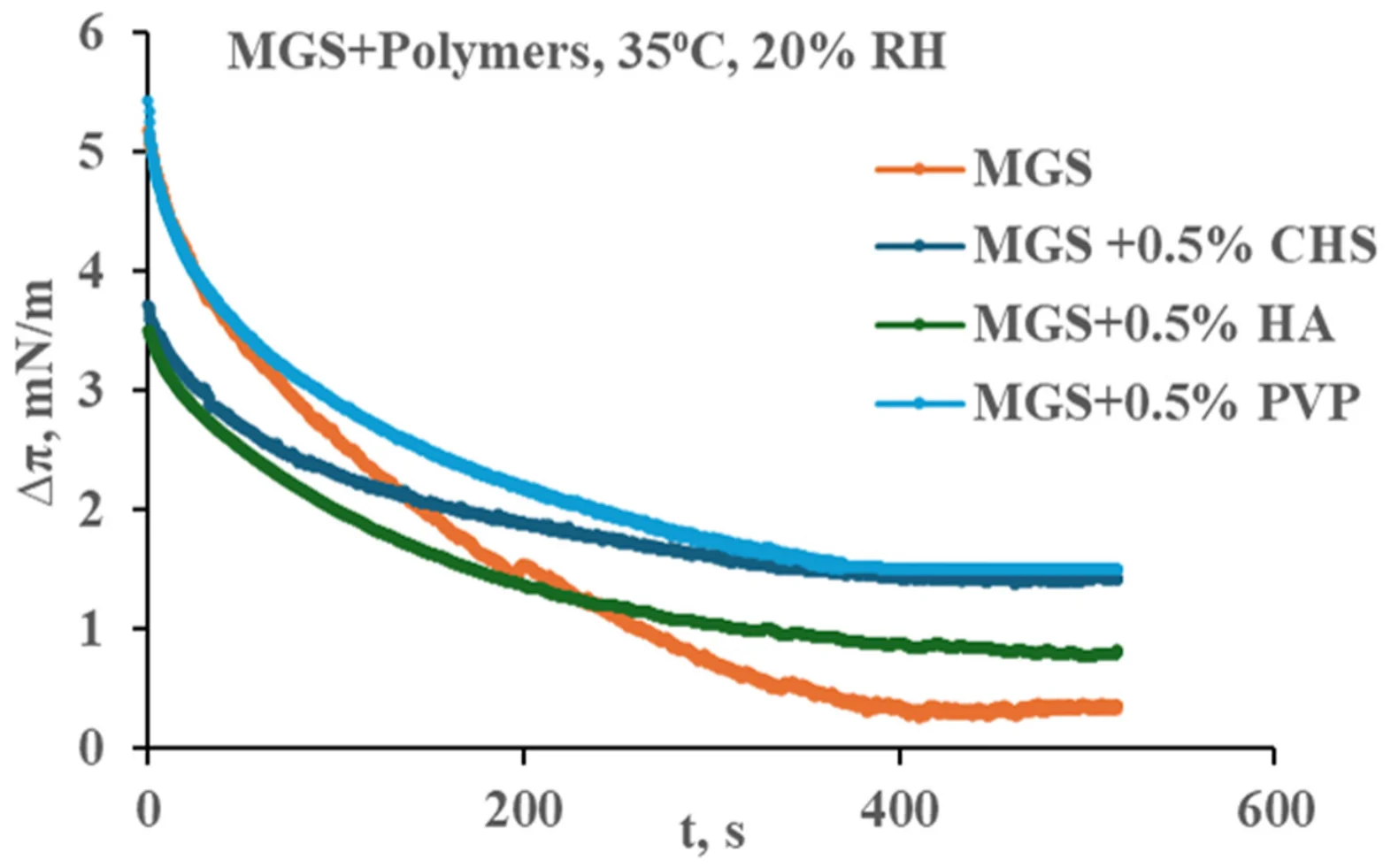
Surface pressure relaxation transients of meibomian films, pure and in the presence of 0.5% mucomimetic polymers, at 35 °C, 20% RH.

The analysis (Table 6) of surface pressure relaxation transients (Figure 12) reveals that inclusion of mucomimetic polymers raises ∆π_inf_ while changing the balance between viscoelastic and diffusion processes.

### 3.4. Surface Properties of Meibum Polymer Films upon Exposure to Microparticulates: 35 °C, 80% RH, PM2.5

**Figure 13:**
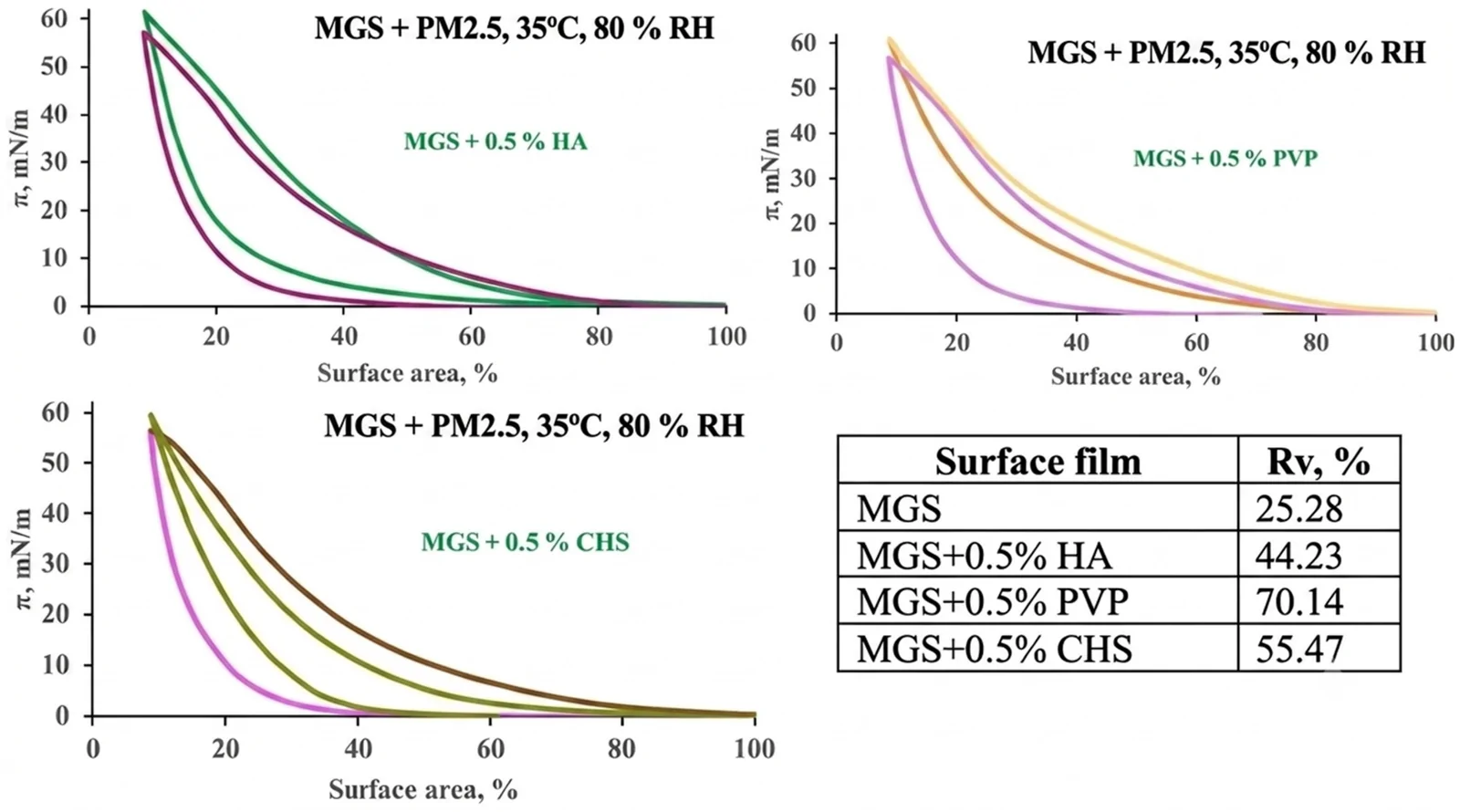
Compression–expansion π(A) isocycles of MGS films, alone (purple line) and upon inclusion of 0.5% mucomimetic polymers in the aqueous subphase at 35 °C, 80% RH, PM2.5. The tabular insert summarizes the isothermal reversibility values as calculated with Equation (8).

**Figure 14:**
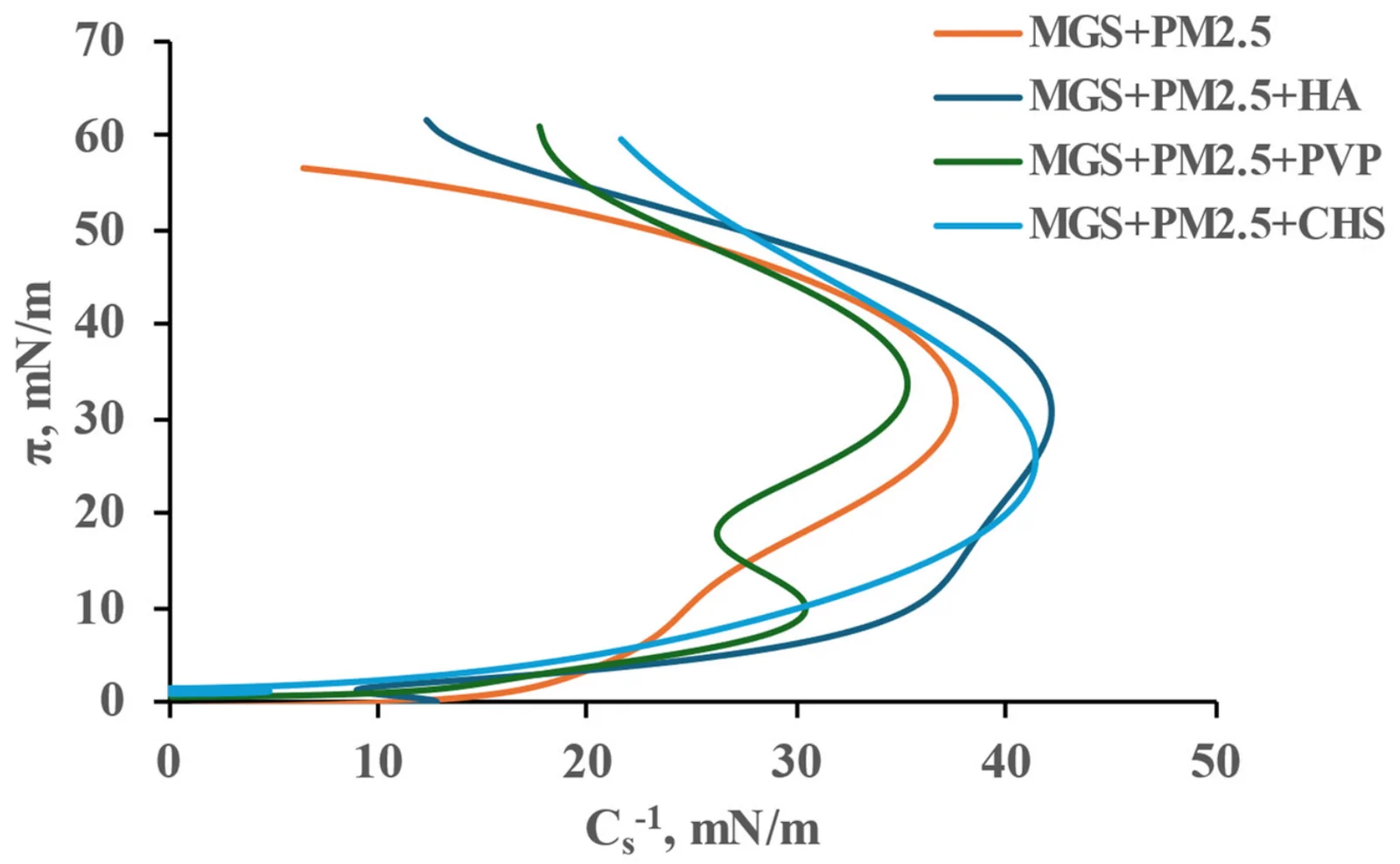
Isothermal reciprocal compressibility modulus/surface pressure dependencies of MGS layers, alone and upon inclusion of 0.5% mucomimetic polymers in the aqueous subphase, at 35 °C, 80% RH, PM2.5.

The introduction of PM2.5 led to a measurable decline in isothermal reversibility. Concurrently, a distinct shift in the π(A) isocycles towards higher surface pressure (Figure 13) compared to surface film behavior at normal ambient conditions. Despite these shifts, the films consistently exhibited a liquid-expanded-like state, characterized by a surface compressibility modulus *C_s_^−^*^1^ < 50 mN/m. Specific points of inflection within the *C_s_^−^*^1^ (π) profiles suggest the occurrence of intricate structural reorganizations within the interfacial layer at specific surface pressure intervals (Figure 14).

Probing the π(A) compression isotherms using Equations (5) and (7) yields the data presented in Table 7, indicating that the presence of polymers induces varied structural transitions in molecular packing at the aqueous-film interface.

**Figure 15:**
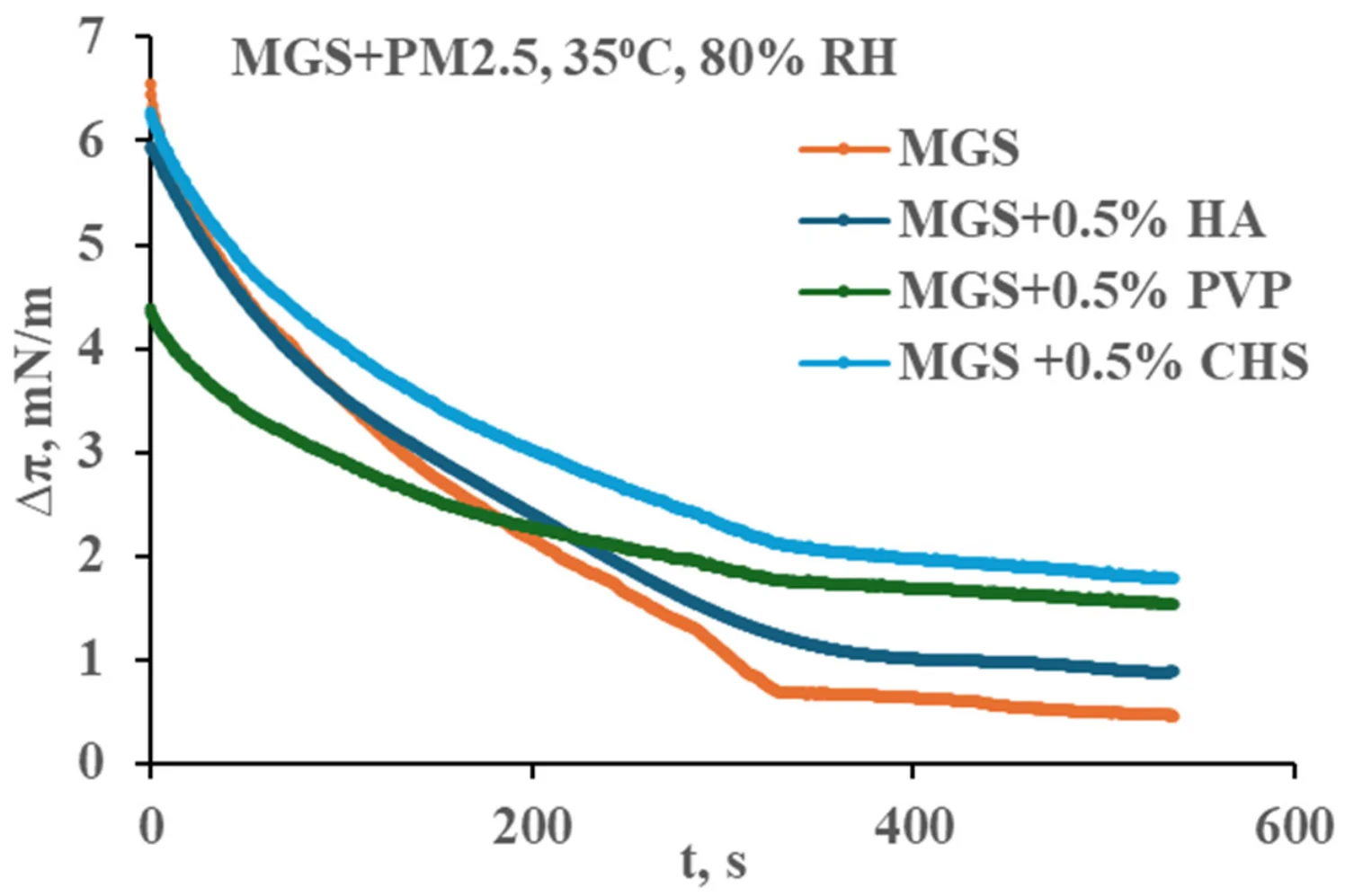
Surface pressure relaxation transients of meibomian films, pure and in the presence of 0.5% mucomimetic polymers, at 35 °C, 80% RH, PM2.5.

Data derived from the surface pressure relaxation transients (Figure 15) and summarized in Table 8 demonstrate that the integration of mucomimetic polymers enhances the ∆π_inf_ values. Furthermore, these additives significantly modify the kinetic equilibrium between viscoelastic relaxation and diffusion-controlled mechanisms within the interfacial layer.

## 4. Discussion

The integrity of the ocular surface and the stability of the tear film are fundamentally dependent on the sophisticated, stratified arrangement (see Figure 1 and Figure 4) of the meibomian gland secretion (MGS) [1,2,39]. This interfacial architecture is characterized by a dual-layered composition: a distal nonpolar region, primarily comprised of cholesterol and wax esters, which is theorized to inhibit the evaporation of the aqueous tear (AT) phase; and a proximal polar lipid layer—rich in (O-acyl)-ω-hydroxy fatty acids and phospholipids—that facilitates the adhesion of the lipid film to the underlying aqueous subphase. Such a highly ordered configuration ensures efficient modulation of surface tension, promotes rapid lateral spreading during the blink cycle, and maintains the viscoelastic flexibility of the film [40]. However, when external stressors—including particulate matter or fluctuations in ambient temperature and humidity—interfere with this molecular alignment, the film’s ability to act as a cohesive barrier is impaired. This structural degradation (as in left panel of Figure 7) results in accelerated tear fluid loss and diminished film persistence. Consequently, maintaining the native multilayered morphology of the MGS is vital for protecting the biophysical and physiological health of the eye.

The current investigation examines the in vitro morphological features, mechanical compressibility (Cs^−1^), and viscoelastic dynamics—determined by the equilibrium between diffusional and viscoelastic contributions to stress relaxation—of lipidic interfaces. By utilizing a Langmuir trough at the air–water interface, this model decouples meibomian gland secretion (MGS) films from auxiliary tear components and ignores physiological variables like tear replenishment, blink rates, and epithelial crosstalk. Although such simplifications may reduce direct translatability to in vivo scenarios, they allow for rigorous experimental standardization. This approach isolates MGS properties from confounding biological factors to identify fundamental correlations with clinical ocular surface performance. Prior studies involving both healthy cohorts and individuals with Dry Eye Disease (DED) substantiate that these laboratory metrics serve as robust proxies for the real-world behavior of the human tear film (TF) and its lipid layer (TFLL) [1,12,41,42,43,44,45,46,47]. Clinical data mirror these in vitro findings: DED-derived samples typically show compromised surfactant functionality and an inability to sustain a stable duplex architecture under compression, whereas healthy controls remain cohesive [1,12]. On a macroscopic scale, the elasticity of the TFLL is evidenced by its restorative capacity post-deformation, specifically quantified through (i) the velocity of lateral spreading following eyelid opening and (ii) the rate of structural attrition during consecutive blink cycles [42,43]. A certain limitation of our study stems from the restriction inherently set by the tiny amount of meibum that can be collected from volunteers, which in turn requires its careful utilization across experiments. Therefore, each polymer (with mean molecular weight 1000 kDa) and each environmental stressor (low RH, low temperature, PM2.5) is tested at a single, fixed condition. The choice of environmental stressors conditions is explained elsewhere [20] while the polymer molecular weight and inclusion at 0.5% are selected based on their current and intended implementation in ophthalmic formulations. It is of significant practical interest and target of future studies to probe the impact of environmental stressors in a broader range as it will allow for assessment of thresholds or nonlinear effects and for predictive understanding of how various levels of these conditions will impact the performance of TFLL. The same is valid for evaluating the influence of varying the molecular weight of polymers, which is known to differentially affect the physicochemical properties and stability of tear film in vivo [48,49,50].

Provided that other parameters remain unchanged, a more elastic Tear Film Lipid Layer (TFLL) is anticipated to undergo more accelerated expansion across the air–aqueous interface following a blink. This spreading phase is clinically indispensable; as the TFLL migrates superiorly, it generates a mechanical traction that draws the underlying aqueous phase upward. This mechanism ensures homogenous corneal lubrication, bolsters tear film stability, and mitigates localized break-up [44,45,46,47,51]. Investigations by Yokoi et al. [43] substantiate that TFLL propagation is notably impaired in subjects with Dry Eye Disease (DED) relative to healthy cohorts. Additionally, Goto and Tseng [46] characterized specific anomalies in patients with Meibomian Gland Dysfunction (MGD): in addition to a significantly delayed spreading duration (3.54 ± 1.86 s vs. 0.36 ± 0.22 s), the expansion morphology lacked horizontal symmetry. Rather than advancing as a uniform linear front, MGD-derived films exhibited vertical striations that migrated at disparate velocities, resulting in a fragmented boundary. Furthermore, the topographical roughness of the lipid layer—a proxy for thickness non-uniformity—was substantially elevated in these individuals (lipid map uniformity = 125 nm^2^) compared to healthy controls (lipid map uniformity = 14 nm^2^) [51]. This correlates with the heightened structural heterogeneity observed (Figure 7) via Brewster Angle Microscopy (BAM) in meibum subjected to environmental stressors [47].

The structural resilience of the Tear Film Lipid Layer (TFLL) can also be gauged by the velocity of its morphological deterioration during the inter-blink interval. Enhanced elasticity generally provides superior structural stability; conversely, research by Georgiev et al. [1,12] indicates that the rate of pattern breakdown is significantly more pronounced in individuals with Meibomian Gland Dysfunction (MGD). This suggests that their lipid interfaces are characterized by a predominant dissipative viscous behavior rather than the robust elastic nature typical of healthy eyes [41]. These observations are corroborated by findings from the Goto research group [46]. Accordingly, the capacity of mucomimetic polymers to bolster the expansion dynamics and structural persistence of meibum films under simulated environmental stressors in vitro acts as a reliable indicator of their clinical effectiveness in vivo [41].

This research indicates that reduced thermal conditions, arid environments, and atmospheric particulates induce analogous deleterious shifts in the interfacial characteristics and structural cohesion of meibomian gland secretion (MGS) layers. These stressors promote heightened film stiffness, perturbed molecular packing, and diminished interfacial stability—factors that collectively undermine the tear film’s defensive capacity [8,9,10,52]. Such observations imply that atmospheric conditions typical of dry or contaminated regions facilitate tear film collapse and contribute to the etiology of evaporative dry eye disease.

The impact of low humidity and cold temperatures aligns with fundamental thermodynamic principles. In low-humidity environments, accelerated evaporation of the aqueous subphase triggers evaporative cooling at the air–lipid boundary [24], potentially decreasing the TFLL temperature by 5–10 °C [53]. This thermal drop shifts the thermodynamic equilibrium, inducing phase transitions toward higher molecular ordering and rigidity [51]. As the lipid layer loses its requisite fluidity, it becomes susceptible to structural failure and less capable of stabilizing the ocular surface [41,54,55,56].

Concurrently, exposure to fine particulate matter, specifically PM2.5 facilitates the aggregation of MGS lipids, resulting in increased film heterogeneity [57,58]. This clustering disrupts homogenous lateral spreading, producing a non-uniform, patchy morphology with compromised durability. While the biochemical pathways of particulate interaction differ from the purely physicochemical effects of temperature and humidity, the macroscopic impact on film integrity is strikingly congruent. Such aggregation likely stems from direct particle–lipid interactions that recalibrate intermolecular forces and promote localized nucleation [59,60]. Precisely defining the mechanistic pathways for PM2.5 remains difficult due to the heterogeneous chemical profile of these aerosols, which include water-soluble ions, inorganic elements, and varied carbonaceous compounds [61]. Specifically, the concentration of polycyclic aromatic hydrocarbons (PAHs)—which are prone to partitioning into the non-polar stratum of the MGS—varies significantly in environmental samples. This variability, alongside fluctuations in metallic or microplastic content, suggests that different particulate compositions may elicit distinct impacts on TFLL functionality compared to those observed in this controlled study.

In the current study, a sophisticated analytical apparatus was developed to analyze the impact of polymers on the surface properties of MGS duplex multilayers: (i) a Volmer equation-based version of 2D-VES (Equation (5)) which (together with Equation (7)) makes it possible to probe the apparent number of molecules located at the MGS/aqueous interface as well as their unique molecular properties (i.e., limiting area per molecule, molecular compressibility, cohesion pressure, etc.) and (ii) a combined Maxwell viscoelastic and diffusion-relaxation model (Equation (10)) to quantify the transient behavior of the dilatational relaxation modulus. Apart from the exposure to decreased RH where a complex picture is observed (probably due to in-depth incorporation of dehydrated polymer chain residues in the MGS films), at most ambient conditions it can be seen that the supplementation of polymers resulted in more compressible (higher value of *k*) films and in a drop of *π_coh_* (i.e., improved spreading) which is typical for polymer-containing films. In line with this, the presence of PVP resulted in an increase of the apparent values of *A_lim_* and decrease of the apparent number of molecules at the film/aqueous interface which is also indicative for the inclusion of polymers in the molecular packing. At 35 °C, 80% RH there is a somewhat unique behavior of CHS which results in an increase of the apparent number of molecules at the MGS/aqueous interface. This suggests a distinct mode of action at normal ambient conditions compared to the rest of the polymers. As per MGS and in many cases MGS + 0.5% CHS layers the *A_lim_* are typical for different degrees of compression of OAHFA, the intrinsic amphiphilic species of MGS.

The analysis of stress relaxation transients (Figure 5, Figure 9, Figure 12 and Figure 15) reveals that mucomimetic polymers altered the relative contributions of viscoelastic and diffusion processes in the films and invariably resulted in higher ∆π_inf_ suggesting enhanced elastic response of the layers upon dilatational deformation. Apart from shifting the relaxation process towards higher ∆π_inf_ and the supplementation of polymers changed the balance between the viscoelastic and diffusion processes with an overall rise of the contribution of viscoelasticity compared to the one of diffusional rearrangement of molecules across the strata of the MGS duplex film. These in vitro results are consistent with documented clinical evidence regarding ocular surface therapeutic interventions. Specifically, the topical administration of 0.1% hyaluronic acid (HA) has been demonstrated to augment the thickness of the tear film lipid layer (TFLL) in lipid-deficient patients, increasing it from sub-60 nm to 75 nm within 15 min post-instillation [15]. Furthermore, these data mirror the observed effects of a 3% diquafosol ophthalmic solution (i.e., a secretagogue known to stimulate rapid MUC5AC mucin release) which has been shown to sustain increased TFLL thickness in healthy subjects for over an hour following application [16]. The observed MGS-modulating properties of water-soluble mucomimetic polymers appear to stem from the establishment of an interfacial, gel-like polymer matrix. This structural assembly is primarily driven by an extensive network of hydrogen bonds occurring both inter-polymerically and between the polymer chains and the polar lipid headgroups. Consequently, this consolidation facilitates elevated film viscosity, enhanced lateral homogeneity of the lipid monolayer, and improved aqueous integration within the tear film structure [1,12]. Operating on a physiologically significant timescale of mere seconds, this rapid kinetic mechanism reveals that the distinct strata of the tear film function not as isolated compartments, but as an integrated, dynamic continuum characterized by perpetual interlayer crosstalk and systemic interdependence.

The biophysical and mechanistic effects are thought to be the primary route of action of polymeric eyedrops in the restoration of the tear film and ocular surface integrity and represent the main focus of the current study. Although the performance of the polymers is certainly dependent on the environmental stressors, still some general patterns are identifiable. Polymers improve the spreading of lipid films, as manifested by the decrease of π_coh_ and of isothermal reversibility. PVP, CHS, and HA facilitate uniform lipid distribution and also partially integrate into the existing film, thereby helping to restore duplex multilayer organization disrupted by low humidity, low temperature, or particulate matter. At 35 °C and 80% RH, CHS shows characteristic effect, resulting in an increase of the apparent number of molecules at the MGS/aqueous interface. This suggests a distinct impact at normal ambient conditions compared to the other polymers. As observed for MGS and, in many cases, MGS + 0.5% CHS layers, the A_lim_ values are typical for different degrees of compression of OAHFA, the intrinsic amphiphilic species of MGS. Also, it should be noted that the presence of HA tended to result in a strong increase of the A_lim_ value and a corresponding decrease in the apparent mean molecular number of molecules (as calculated with Equations (5) and (7)), which suggests a distinct mode of interaction of HA with MGS films. The results, as well as the multiple numerical estimates obtainable via the approaches described here, may be beneficial for the design of artificial tears for the treatment of dry eye. To draw a definitive conclusion, it is necessary to establish statistical correlations between the in vitro estimates and the in vivo clinical performance of the eyedrop formulations, which is a subject of our subsequent research.

Apart from the biophysical and mechanistic implementations, the biochemical interactions may also significantly contribute, especially in cases where long-term residence time of the polymers at the ocular surface is achievable [41]. High-molecular-weight hyaluronic acid (HA) variants have undergone extensive research due to their widespread formulation in lubricating eye drops designed for dry eye therapy [62]. The substantial chain length and viscosity of HA not only bolster corneal and conjunctival hydration but also lower shear stress against the eyelid wiper during blinking [62]. Concurrently, HA mitigates inflammatory responses and downregulates immune activation, which accelerates ocular tissue regeneration [63]. In vitro and in vivo models demonstrate that HA scavenges reactive oxygen species and offers cytoprotection by buffering the cellular toxicity of common ophthalmic preservatives [64,65]. These physiological responses are mediated by transmembrane signaling cascades triggered when HA binds to an array of specific cellular receptors, including CD44, lymphatic vessel endothelial receptor 1 (LYVE-1), receptor for hyaluronan-mediated motility (RHAMM), and toll-like receptor 4 (TLR-4) [66]. Through these receptor interactions, HA signaling critically modulates angiogenesis by directing endothelial cell dynamics [63]. Crucially, the biological outcome of these pathways is highly sensitive to the polymer’s molecular weight. While large HA chains (Mw ≥ 200 kDa) promote cell survival and tissue homeostasis, fragmented, low-molecular-weight HA behaves as a strong mediator of inflammation. This phenomenon is attributed to the different manner of interaction with the HA receptors, CD44 and RHAMM in particular [67]. It is thought that these diverse effects stem from the impact of the molecular weight on the HA conformation: large >MDa HA is a random coil, while very small (e.g., 10 kDa) HA behaves like a rod. Size exclusion chromatography and multiangle light scattering studies revealed that HA mass-to-diameter ratio showed a transition in the 150–250 kDa size range (~65 nm). Hence, the HA rod-to-coil transition occurs in the size range that specifically activates cell signaling by the receptors discussed above. Thus, size-specific signaling could be due to (i) unique external receptor/HA conformation changes enabling transmembrane-mediated activation of cytoplasmic domains, or (ii) transition-size HA may enable multiple receptors to bind the same HA, creating new internal signal-competent cytoplasmic domain complexes [68,69]. Although relatively less studied, similar effects are reported for chondroitin sulfate, demonstrating its (i) high biocompatibility and biodegradability, (ii) propensity to create a favorable environment for tissue repair by reduction of inflammatory processes and oxidative stress, and (iii) binding to CD44 and syndecan cell receptors, enhancing cellular uptake and activating pathways for cell migration, differentiation, and proliferation [70]. While PVP biochemical activity is generally considered limited, it forms a protective polymeric shield over the ocular surface that preserves epithelial integrity by physically sealing micro-gaps between damaged cells. Furthermore, when combined, PVP and HA work synergistically to reduce perilimbal conjunctival erythema (redness) and optimize epithelial repair better than either polymer alone, a cooperative pattern that may be observed with other polymer combinations as well [71,72,73,74,75,76,77,78].

## 5. Conclusions

In conclusion, mucomimetic polymers represent a promising therapeutic approach to partially suppress the detrimental effects of environmental stressors on the structural and surface properties of meibomian gland lipid multilayers. By closely approximating the physicochemical characteristics of the secretory mucins of the natural tear film, these polymers provide a stabilizing subphase that, through complex interactions involving hydrogen bonding with the polar lipid headgroups and partial incorporation of the polymer moieties at the film/aqueous interface, reinforces the lipid layer, enhances its fluidity, and replenishes its interfacial characteristics. PVP, CHS, and HA facilitate uniform lipid distribution and also partially integrate into the existing film, thereby helping to restore multilayer organization disrupted by low humidity, low temperature, or particulate matter. Continued optimization of polymer-containing artificial tears composition and interfacial properties holds potential for developing more robust interventions to preserve visual comfort and eye health in adverse environmental conditions.

## Figures and Tables

**Table 1:** Values of the apparent number of lipid (and polymer) molecules, molecular compressibility, limiting molecular area, and cohesive surface pressure of MGS films, alone or in the presence of 0.5% mucomimetic polymers at 35 °C, 80% RH.

Film	N, Molecules	A_lim_, Å^2^	k, m/mN	π_coh_, mN/m
MGS	4.41 × 10^15^	51.2	0.029	2.914
MGS + 0.5% CHS	6.3 × 10^15^	25.8	0.036	2.724
MGS + 0.5% PVP	2.2 × 10^15^	138	0.0324	1.99
MGS + 0.5% HA	4.55 × 10^14^	454.8	0.0373	0.034

**Table 2:** Fitting parameters obtained by the analysis of stress relaxation transients (Figure 5) via Equation (10) (R^2^ ≥ 0.98).

Surface Film	∆π_inf_, mN/m	∆π_max_, mN/m	A_M_	A_D_	τ_M_. s	τ_D_. s
MGS	0.01	3.941	0.15	0.85	692.6	578.19
MGS + 0.5% CHS	1.6	3.96	0.36	0.64	208.56	89.36
MGS + 0.5% PVP	4.48	4.971	0.98	0.02	176	4.5 × 10^4^
MGS + 0.5% HA	0.3	5.876	0.18	0.82	690.33	475.92

**Table 3:** Values of the apparent number of lipid (and polymer) molecules, molecular compressibility, limiting molecular area, and cohesive surface pressure of MGS films, alone or in the presence of 0.5% mucomimetic polymers at 20 °C, 80% RH.

Film	N, Molecules	A_lim_, Å^2^	k, m/mN	π_coh_, mN/m
MGS	8.02 × 10^15^	24.5	0.028	7.04
MGS + 0.5% CHS	2.9 × 10^15^	163	0.026	2.26
MGS + 0.5% PVP	8.88 × 10^14^	679	0.051	0.504
MGS + 0.5% HA	9.32 × 10^14^	599.14	0.0248	0.0126

**Table 4:** Fitting parameters obtained by the analysis of stress relaxation transients (Figure 9) via Equation (10) (R^2^ ≥ 0.98).

Surface Film	∆π_inf_, mN/m	∆π_max_, mN/m	A_M_	A_D_	τ_M_. s	τ_D_. s
MGS	0.1	4.362	0.89	0.11	574.95	20.18
MGS + 0.5% CHS	1.2	5.08	0.47	0.53	145.36	131
MGS + 0.5% PVP	2	4.46	0.65	0.35	178.9	305.5
MGS + 0.5% HA	1.86	3.871	0.84	0.16	108.39	867.78

**Table 5:** Values of the apparent number of lipid (and polymer) molecules, molecular compressibility, limiting molecular area and cohesive surface pressure of MGS films, alone or in the presence of 0.5% mucomimetic polymers at 35 °C, 20% RH.

Film	N, Molecules	A_lim_, Å^2^	k, m/mN	π_coh_, mN/m
MGS	6.39 × 10^14^	882.7	0.016	1.09
MGS + 0.5% CHS	4.8 × 10^15^	59	0.0417	1.55
MGS + 0.5% PVP	1.32 × 10^16^	19	0.002	6.96
MGS + 0.5% HA	1.17 × 10^16^	20	0.001	6.68

**Table 6:** Fitting parameters obtained by the analysis of stress relaxation transients (Figure 12) via Equation (10) (R^2^ ≥ 0.98).

Surface Film	∆π_inf_, mN/m	∆π_max_, mN/m	A_M_	A_D_	τ_M_. s	τ_D_. s
MGS	0.12	5.186	0.906	0.094	156.96	1.85
MGS + 0.5% CHS	1.012	3.721	0.512	0.408	158.33	208.23
MGS + 0.5% PVP	1.18	5.44	0.64	0.36	156.72	25.18
MGS + 0.5% HA	0.63	3.504	0.83	0.17	151.37	60.3

**Table 7:** Values of the apparent number of lipid (and polymer) molecules, molecular compressibility, limiting molecular area and cohesive surface pressure of MGS films, alone or in the presence of 0.5% mucomimetic polymers at 35 °C, 80% RH, PM2.5.

Film	N, Molecules	A_lim_, Å^2^	k, m/mN	π_coh_, mN/m
MGS	7.75 × 10^15^	73	0.025	7.74
MGS + 0.5% CHS	1.17 × 10^15^	450	0.022	0.047
MGS + 0.5% PVP	2.66 × 10^15^	280.6	0.024	2.8
MGS + 0.5% HA	1.09 × 10^15^	620.9	0.019	1.08

**Table 8:** Fitting parameters obtained by the analysis of stress relaxation transients (Figure 15) via Equation (10) (R^2^ ≥ 0.98).

Surface Film	∆π_inf_, mN/m	∆π_max_, mN/m	A_M_	A_D_	τ_M_. s	τ_D_. s
MGS	0.08	6.554	0.92	0.08	176.74	1.51
MGS + 0.5% CHS	0.57	6.278	0.62	0.38	172.8	1308.15
MGS + 0.5% PVP	0.6	4.377	0.51	0.48	162.5	1384.25
MGS + 0.5% HA	0.47	5.952	0.97	0.03	184.96	1.52

## Data Availability

The data presented in this study are available in the article. Further inquiries can be directed to the corresponding author.

## Abbreviations

The following abbreviations are used in this manuscript:

AT	Aqueous Tear
DED	Dry Eye Disease
MGS	Meibomian Gland Secretion
HA	Hyaluronic Acid
PVP	Polyvinylpyrrolidone
CHS	Chondroitin Sulfate
PM2.5	Particulate Matter (2.5 μm)
TF	Tear Film
TFLL	Tear Film Lipid Layer
